# Medicare for All: A Health Insurance Literacy Perspective

**DOI:** 10.3928/24748307-20210908-01

**Published:** 2021-10

**Authors:** Suad Ghaddar

Health policy proposals are generally evaluated based on various criteria including fiscal feasibility, political viability, ethical and equity considerations, and compatibility with constitutional guiding principles (e.g., personal choice, roles of states vs. the federal government), among others. Throughout the history of health care reform in the United States, the debate has been framed by a combination of the above criteria. Rarely, however, have we considered a health policy proposal from a health literacy, and more specifically, from a health insurance literacy perspective.

Health insurance literacy is defined as “the degree to which individuals have the knowledge, ability, and confidence to find and evaluate information about health plans, select the best plan for their own (or their family's) financial and health circumstances, and use the plan once enrolled” (Quincy, 2012, p. 7). The roll out of the Patient Protection and Affordable Care Act brought the issue of health insurance literacy to the forefront. As millions of people attempted to obtain health care coverage, the complexity of the system became apparent (Bhargava & Loewenstein, 2015) and the need for significant education in health insurance concepts (premiums, deductibles, copayments, coinsurance) was deemed essential for people to make optimal choices and effectively use their newly-acquired health insurance (Blumberg et al., 2013; Loewenstein et al., 2013; Norton et al., 2014). This need was especially pronounced among the previously uninsured, those with low educational attainment, and those from racial and ethnic minority groups, many of whom were navigating the system for the first time (Blumberg et al., 2013); most recent data reveal that significant sociodemographic disparities in health insurance literacy persist (Edward et al., 2019; Villagra et al., 2019). The importance of health insurance literacy is further highlighted by research indicating the negative impact low health insurance literacy has on awareness of health reform efforts (Ghaddar et al., 2018), on enrollment (Hoerl et al., 2017), on plan choice (Braun et al., 2018), on enrollment experiences (Hero et al., 2019), on health care utilization patterns (James et al., 2020; Smith et al., 2018; Tipirneni et al., 2018), on material and behavioral financial hardship (Williams et al., 2020; Zhao et al., 2019), and most likely on health outcomes, although the latter has not yet been empirically evaluated.

Enter Medicare for All. The proposal, apart from the political and fiscal concerns raised using traditional assessment criteria, is promising from a health insurance literacy perspective. Its key advantage is instant name recognition and association with a well-established, favorably perceived program. In its simplicity, it relieves the consumer from many of the challenges faced due to inadequate health insurance knowledge and literacy. As proposed by U.S. Senator Bernie Sanders, the Medicare for All Act (2019) has no complex eligibility criteria (only being a resident of the U.S.), no complicated enrollment processes (program enrollment is automatic upon birth or upon establishing residence in the U.S.), no plan options to choose from (single-payer system), no confusion surrounding deductibles, coinsurance, copayments, (in general, the plan has no cost-sharing provisions), no confusion about in- and out-of-network providers (such a program would most likely have an extensive provider network allowing considerable freedom in choosing a health care provider), and no thorny list of covered/non-covered medical conditions (the plan covers an extensive list of physical health, mental health, vision, and dental benefits, along with some long-term care benefits). However, as the 2020 democratic presidential candidates embraced Medicare for All to mean different things, the initial health-insurance-literacy friendly appeal of Medicare for All started to fade as confusion rose with the unclear positions candidates were taking and the advent of different proposals under variations of the same title: Medicare X, Medicare for America, Medicare Buy-In, Medicare for All Who Want It, Medicare for More. National polls reflected that confusion. While there seemed to be support for a Medicare for All program, that support shifted once people realized what the program entailed (e.g., elimination of private health insurance) (Kirzinger et al., 2019). Additionally, many believed that people would continue to pay deductibles and co-pays when they used health care services and that private insurance plans could be kept (Lopes et al., 2020). Such misconceptions underscored the important role that health insurance literacy plays in the evaluation of candidates' health care reform platforms; adequate levels of health insurance literacy imply that people are asking the right questions and examining the relevant features of a health care proposal.

As current and future presidential, senate, congressional, and local candidates continue discussions on how to expand health care coverage to more Americans, the following are key health insurance literacy guiding principles to consider when proposing health reform legislation: (1) Keep the sociodemographic profile (educational attainment, ethnicity, access challenges) of the U.S. population, including the uninsured, in mind to ensure that the new legislation's demands and complexity level are aligned with the skills and abilities of all consumers, particularly those seeking insurance for the first time. Despite significant gains in health care coverage since the passage of the Affordable Care Act, racial and ethnic minorities and low-income people continue to be overrepresented among the country's uninsured (Berchick et al., 2019; Collins et al., 2016); (2) Apply health literacy principles to health care legislation and its processes. These include, among others, appropriate, actionable, and easy to understand communication as well as representation of the uninsured in planning, implementing, disseminating, and evaluating health care coverage information (Centers for Disease Control and Prevention, n.d.); (3) Develop standardized plan options to minimize the confusion surrounding the many nuanced differences between plans. Comparing a large number of plans with many moving parts (different premiums, different number of in-network providers across various cost-sharing levels) is a daunting experience for many people (Bhargava & Loewenstein, 2015). Recent changes that drop simple choice plans and eliminate requirements for insurers to provide meaningfully different plans represent a move in the opposite direction to simplification of the choice process (Collins, 2018); (4) Complement internet-based application platforms with online decision support tools. Such tools have shown promise in improving the quality of health insurance decisions (Bundorf et al., 2019; Politi, Barker, Kaphingst, et al., 2016; Politi, Kuzemchak, Liu, et al., 2016); and (5) Fund enrollment and post-enrollment consumer assistance programs to support individuals learning how to obtain and use health insurance for the first time. The Affordable Care Act, despite its initial implementation challenges, was cognizant of the need for consumer assistance. For example, it authorized various navigator and non-navigator assistance programs followed by millions of dollars in grants to provide outreach, education, and assistance to consumers (Centers for Medicare & Medicaid Services, 2019; Reichard, 2013). Under the previous administration of President Donald J. Trump, these programs have suffered significant budget cuts, decreasing from a high of $63 million in 2017 to just $10 million annually in 2020 and 2021 (Keith, 2019).

Clearly, system-level changes are slow and difficult to implement. That's why advocacy, an increasingly central area of responsibility for health educators and professionals (Accreditation Council for Graduate Medical Education, 2020; Hubinette et al., 2017; National Commission for Health Education Credentialing, 2019), is essential to move such efforts forward. Health educators and professionals play an important role in advocating for a simpler, easier to understand health insurance system that empowers consumers to make informed health coverage decisions in support of their and their families' health care needs. Lessons can be learned from advocacy efforts targeting health literacy. Such efforts have been successful at integrating health literacy in Healthy People 2010, 2020, and 2030 goals (Advisory Committee for Healthy People 2030, 2018; Office of Disease Prevention and Health Promotion, n.d.; U.S. Department of Health and Human Services, 2000), at launching a National Action Plan to Improve Health Literacy (U.S. Department of Health and Human Services, 2010), at establishing dedicated federal funding (e.g., National Institutes of Health) to support research that furthers understanding of health literacy, and at having a Roundtable on Health Literacy to maintain discussion and dissemination of research findings and best practice recommendations (The National Academies of Sciences, Engineering, and Medicine, n.d.). A similar advocacy effort for health insurance literacy has the potential to galvanize stakeholders to support health-insurance-literate plans at the private sector level and ultimately health-insurance-literate legislation at the public sector level. Along the way, health education and promotion specialists can advance health insurance literacy by developing and delivering educational programs through academic extension programs, adult education venues, and public health outreach settings. Such programs, albeit on a limited scale, have shown promise in increasing individual-level knowledge and skills and improving the capacity to make optimal health insurance purchases (Brown et al., 2016; Brown, 2018; Patel et al., 2019).

A Medicare for All system is certainly not a panacea from a health insurance literacy perspective. Even if such a plan were implemented, some access and utilization challenges stemming from inadequate health insurance literacy are likely to persist. A large system is bound to be complex and will present its own comprehension and navigation challenges. Evidence from the current Medicare program reveals that enrollees struggle with identifying the health coverage they have (e.g., Medicare vs. Medicare Advantage) and how it works (Huffman & Upchurch, 2018), and that poorer familiarity with the program translates into delays and avoidance of seeking care (Morgan et al., 2008). Similarly, evidence from other countries reveals that a national health coverage plan does not eliminate health insurance literacy issues. Universal coverage health systems, by virtue of their size, are complex and require a high level of health insurance literacy skills to navigate; they also evolve over time to incorporate additional features each with its own jargon and complexities. In Israel, for example, the national health insurance system is complemented by supplementary health insurance; evidence reveals gaps in knowledge and understanding of available coverage options resulting in suboptimal purchase and utilization patterns (Green et al., 2017). In France, where college students are required to purchase a compulsory health insurance plan with the option of a complementary plan, health insurance literacy remains a challenge given the wide range of specific conditions, modalities, and costs that vary based on each student's personal circumstance (Montagni et al., 2018).

Irrespective of its contentious features as well as its political, fiscal, and implementation challenges, Medicare for All, in its pure form, brings our attention to a new ideal to strive for—a health literacy sensitive health legislation that promises a simplified insurance process supporting improved decision-making when selecting health care coverage and using health care services. As future leaders formulate the details of their health care proposals and/or plan for the reform of existing laws, it is imperative to strive for such an ideal, most importantly to ensure that such laws help those they aim to expand coverage to—the uninsured, the vulnerable, and the marginalized.
